# Efficacy, safety, and strategy-modifying factors of pulsed field ablation for persistent and long-standing persistent atrial fibrillation: a systematic review and meta-analysis

**DOI:** 10.3389/fcvm.2026.1877204

**Published:** 2026-10-05

**Authors:** Yuanlin Zhou, Jianhua Xie, Mingliang Fang, Shipeng Wang, Xiaohong Bin, Qingyao Liao

**Affiliations:** Department of Cardiology, West China School of Medicine, Sichuan University, Sichuan University Affiliated Chengdu Second People's Hospital, Chengdu Second People's Hospital, Chengdu, Sichuan, China

**Keywords:** long-standing persistent atrial fibrillation, meta-analysis, persistent atrial fibrillation, posterior wall isolation, pulsed field ablation, systematic review

## Abstract

**Background:**

Pulsed field ablation (PFA) is increasingly applied in persistent atrial fibrillation (PerAF) and long-standing persistent atrial fibrillation (LSPAF). Previous reviews of mixed AF populations suggested potential advantages in procedural efficiency and esophageal safety, but PerAF/LSPAF-specific evidence on long-term rhythm outcomes and on the value of posterior wall or extended substrate strategies remains limited, with substantial heterogeneity in lesion sets, endpoint definitions, and comparator types.

**Methods:**

We searched PubMed, Web of Science, Embase, Cochrane Library, and Scopus through April 18, 2026, with an updated verification search performed in August 2026. Because of clinical and methodological heterogeneity, formal pairwise pooling was restricted to comparisons supported by at least two clinically homogeneous head-to-head studies; single-study comparisons were synthesized narratively, and strategy-level outcomes were explored with single-arm proportion meta-analysis of exact-count cohorts. Off-antiarrhythmic drug (off-AAD) outcomes were prioritized when available. Certainty of evidence was rated using the GRADE approach.

**Results:**

Twenty-two studies (approximately 3,100 patients) were included. In an exploratory pooled analysis of the only two available head-to-head comparisons (380 patients, with imbalanced study weights and different lesion sets: PVI-only vs. PVI plus posterior wall isolation), PFA was associated with lower 12-month atrial arrhythmia recurrence than cryoballoon ablation [risk ratio [RR] 0.71, 95% confidence interval [CI] 0.51–0.98; GRADE certainty: very low]; this is best regarded as a preliminary signal. No statistically detectable difference in major complications was observed between PFA and cryoballoon [risk difference (RD) 0.00, 95% CI −0.02 to 0.02], reflecting absence of a demonstrable difference rather than proven equivalence. Single-study comparisons against radiofrequency ablation (RR 0.65, 95% CI 0.45–0.94) and hybrid-convergent ablation (RR 1.11, 95% CI 0.67–1.86) yielded directionally discordant results and are presented narratively. Exploratory, non-comparative single-arm analyses yielded pooled 12-month freedom from atrial arrhythmia of 66.0% (95% CI 55.5%–75.2%; I²=79.0%) for PVI plus posterior wall cohorts and 82.6% (95% CI 76.3%–87.5%; *I*^2^ = 0%) for beyond-PV/extended substrate cohorts; these estimates derive from different, non-randomized cohorts and are hypothesis-generating only.

**Conclusions:**

Current evidence suggests favorable efficacy and safety of PFA in PerAF/LSPAF, but certainty is low to very low for all key outcomes. The lower recurrence vs. cryoballoon ablation is an encouraging but preliminary signal requiring confirmation in adequately powered randomized trials with uniform lesion sets. Whether extended substrate strategies improve outcomes cannot be determined from current non-comparative data. Future studies should apply standardized off-AAD endpoints, uniform rhythm monitoring, and stratification by atrial myopathy burden and PFA platform.

**Systematic Review Registration:**

identifier INPLASY202640082; doi: 10.37766/inplasy2026.4.0082

## Introduction

Persistent atrial fibrillation (PerAF) and long-standing persistent atrial fibrillation (LSPAF) are associated with worse rhythm outcomes after catheter ablation than paroxysmal AF, largely because of more advanced atrial electrical and structural remodeling. Whether ablation strategies extending beyond pulmonary vein isolation (PVI) improve outcomes in these patients remains one of the most debated questions in AF ablation.

Pulsed field ablation (PFA), a non-thermal and predominantly myocardial-selective energy source, has demonstrated potential advantages in procedural efficiency and collateral-tissue safety relative to thermal ablation (1–4), making it an attractive candidate for strategies that extend beyond PVI. The mechanistic rationale is strongest for the left atrial posterior wall, which shares a common embryological origin with the pulmonary veins; posterior wall fibrosis, conduction anisotropy, and septo-pulmonary bundle-related electrical remodeling may all contribute to the initiation, maintenance, and recurrence of persistent AF (5). Posterior wall isolation and more extensive substrate modification have consequently long been regarded as candidate strategies for PerAF treatment (6–8).

However, the hypothesis that more extensive ablation yields better outcomes lacks consistent randomized support. The STAR AF II trial found no benefit of routinely adding linear or complex fractionated atrial electrogram ablation to PVI in persistent AF (9); the CAPLA trial found no reliable reduction in recurrence from routine posterior wall isolation at first ablation (10); and DECAAF II found no net benefit of empiric fibrosis-guided substrate ablation without rigorous patient selection (11). Meta-analyses of posterior wall strategies in the thermal era are likewise discordant: Shrestha et al. suggested that PVI plus posterior wall isolation may reduce recurrence (6), whereas randomized-trial-focused analyses by Ibrahim et al. and Li et al. found no consistent benefit and a possible increase in atrial tachycardia/flutter recurrence (7, 8). A focused review of pentaspline-catheter PFA for posterior wall isolation by Pranata et al. demonstrated high technical feasibility and reassuring acute esophageal safety but no confirmed recurrence benefit (12).

Meanwhile, existing meta-analyses comparing PFA with thermal ablation have mostly pooled paroxysmal and persistent AF and centered on first-time PVI (1–4). They consistently indicate shorter procedure times and lower esophageal injury risk with PFA, but recurrence advantages are unstable (1, 4), and increased cardiac tamponade signals or prolonged fluoroscopy have been noted in some analyses (3, 4). Because these reviews mixed AF types and lesion sets, they cannot answer whether, specifically in PerAF/LSPAF, PFA compares favorably with established ablation technologies or whether the lesion-set strategy modifies PFA outcomes.

We therefore conducted this systematic review and meta-analysis restricted to PerAF/LSPAF to address three structured questions: (1) How does PFA compare with established ablation technologies (cryoballoon, radiofrequency, and hybrid-convergent ablation) in 12-month rhythm outcomes and major complications? (2) What is the safety profile of PFA in this population, including emerging PFA-specific complications? (3) Do reported outcomes differ across lesion-set strategies (PVI-only, PVI plus posterior wall isolation/ablation, and beyond-PV extended substrate ablation)? Because substantial clinical and methodological heterogeneity was anticipated, we pre-specified a stratified analytical framework: formal pairwise meta-analysis only where at least two clinically homogeneous head-to-head studies addressed the same comparator and outcome, and narrative or exploratory single-arm synthesis otherwise.

## Methods

### Protocol and registration

This systematic review and meta-analysis was conducted and reported in accordance with the Preferred Reporting Items for Systematic Reviews and Meta-Analyses (PRISMA) 2020 guidelines (13). The review protocol was registered with the International Platform of Registered Systematic Review and Meta-Analysis Protocols (INPLASY; registration no. INPLASY202640082 on 24 April 2026). In the interest of full transparency, we emphasize that this registration was performed retrospectively, after completion of the literature search. Retrospective registration does not mitigate the absence of prospective registration, is not presented as equivalent to it, and is acknowledged as a methodological limitation (see Limitations).

### Search strategy

We systematically searched PubMed, Web of Science, Embase, Cochrane Library, and Scopus from inception through April 18, 2026. The search strategy combined terms related to pulsed field ablation, persistent atrial fibrillation, and long-standing persistent atrial fibrillation (complete search strategy provided in Supplementary Appendix). No language restrictions were applied. Additionally, reference lists of included studies and relevant review articles were hand-searched to identify potentially eligible studies. An updated verification search was performed on August 17, 2026, during revision, to identify any eligible studies published or indexed since the primary cutoff; this process formally re-evaluated two additional reports [ADVANTAGE AF Phase 2 (42) and MANIFEST-US 43], whose disposition is described in the Results.

### Eligibility criteria

Studies were eligible if they: (1) enrolled adult patients (≥18 years) with PerAF or LSPAF; (2) evaluated PFA as the intervention; and (3) reported interpretable efficacy and/or safety outcomes. We included randomized controlled trials (RCTs), prospective cohort studies, retrospective comparative studies, and large registry-based analyses. Conference abstracts, case reports, editorials, and studies without extractable numerical data were excluded. Studies enrolling mixed paroxysmal/persistent AF populations without separately extractable PerAF/LSPAF outcome data were excluded at full-text review; this was the pre-specified reason for excluding the MANIFEST-US safety survey from formal synthesis (43).

### Terminology and strategy definitions

The terms PWI, LAPWI, and PWA are not strictly interchangeable and are defined here explicitly. Posterior wall isolation (PWI) denotes electrical isolation of the left atrial posterior wall confirmed by entrance and/or exit block; left atrial posterior wall isolation (LAPWI) is used synonymously with PWI when confirmed isolation was reported. Posterior wall ablation (PWA) denotes delivery of ablation lesions to the posterior wall without universal, systematic confirmation of durable isolation. Because source studies used these terms inconsistently, we grouped PWI/LAPWI/PWA into a single strategy tier (“PVI plus posterior wall isolation/ablation”) for synthesis, while acknowledging that they are not identical procedures; this grouping is itself a source of clinical heterogeneity and is considered in the interpretation.

### Study selection and data extraction

Two reviewers independently screened titles and abstracts assessed full-text articles and extracted data using a standardized form. Disagreements were resolved through discussion or consultation with a third reviewer. Extracted data included: study design, population characteristics (sample size, AF type, baseline characteristics), PFA strategy and lesion set, PFA catheter platform and energy system, comparator type (if applicable), follow-up duration, blanking period definition, AAD management approach, primary efficacy endpoint definition, and safety outcomes.

### Outcome definitions

#### Primary efficacy endpoint

Atrial arrhythmia recurrence after the blanking period at approximately 12 months, or its study-level equivalent. When studies reported both off-AAD and on-AAD results, the off-AAD outcome was prioritized. When AAD status was not explicitly separated, the study-level primary rhythm endpoint was included as originally reported and labeled as AAD-unseparated. Composite efficacy endpoints incorporating AAD escalation (e.g., pivotal IDE studies) were retained for descriptive synthesis only and excluded from precise-count single-arm pooling.

#### Safety endpoint

Major complications were first extracted as reported in original studies, then mapped to current Heart Rhythm Society/European Heart Rhythm Association/Expert Consensus on Catheter and Surgical Ablation (HRS/EHRA/ECAS) standards: events requiring invasive management or escalated therapy, prolonged hospitalization, clinically significant hemodynamic instability, stroke/systemic embolism, myocardial infarction, clinically significant cardiac tamponade, major vascular complications requiring intervention or transfusion, or events resulting in persistent disability or death.

### Quality assessment and certainty of evidence

Comparative cohorts were assessed using the Newcastle–Ottawa Scale (NOS). Single-arm and registry studies were evaluated using a modified 7-item tool assessing: cohort representativeness, population definition, strategy definition, endpoint clarity, follow-up adequacy, safety reporting, and completeness/loss to follow-up (detailed scoring criteria in Supplementary Table S1). Study-level quality scores are presented in Table 4 and Figure 1. In addition, the certainty of evidence for each key outcome was rated using the GRADE (Grading of Recommendations Assessment, Development and Evaluation) approach, considering risk of bias, inconsistency, indirectness, and imprecision; evidence from non-randomized studies starts at low certainty and was downgraded where appropriate (Supplementary Table S3).

**Figure 1:**
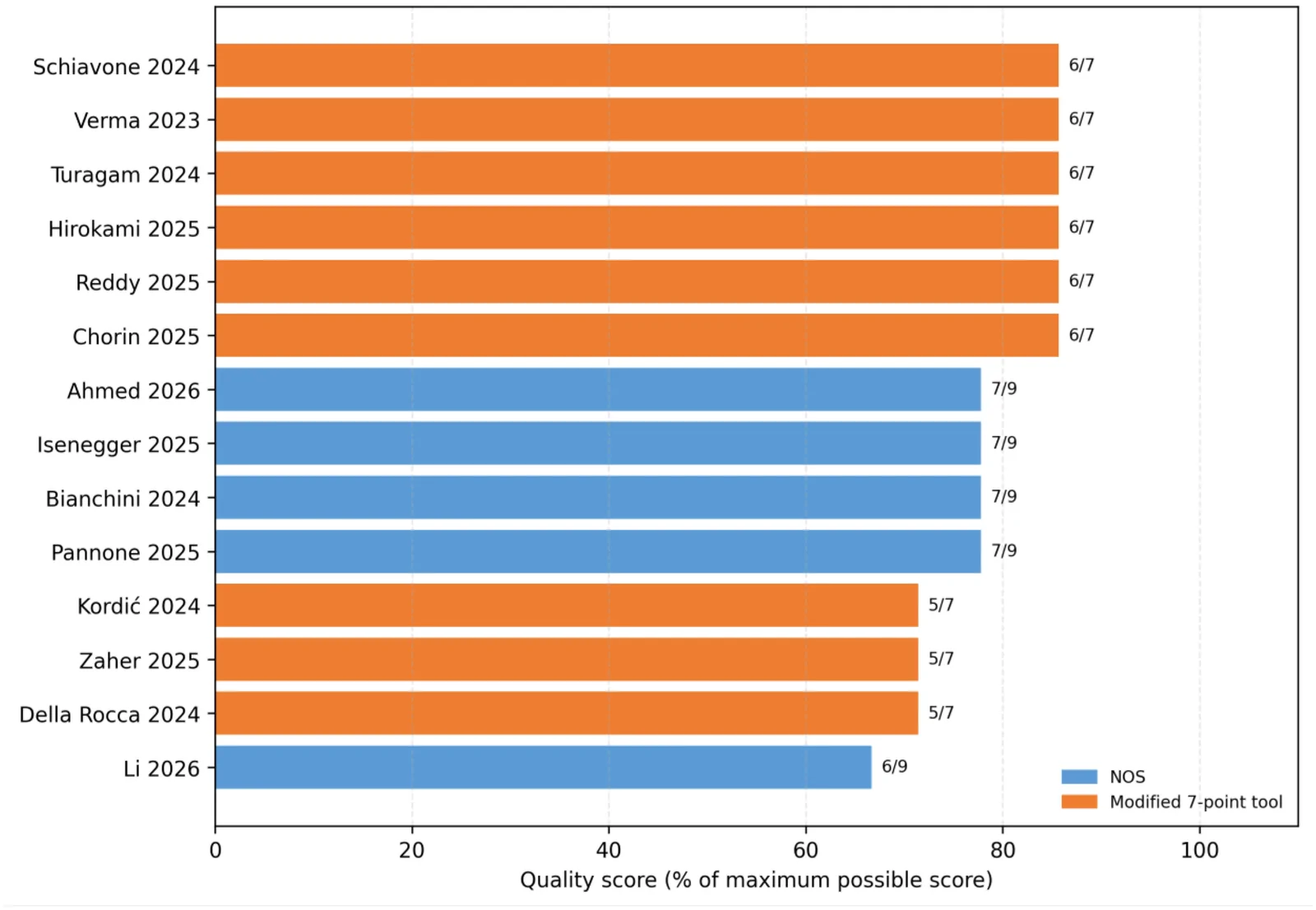
Study quality assessment for the main-study set (scores expressed as percentage of maximum possible score). Scores are shown as percentage of the maximum possible score so that NOS and the modified 7-point tool can be displayed on a common scale. The figure summarizes the 14 main-study reports listed in Table 1 rather than every background-only report in the systematic review.

**Table 1:** Characteristics of all 22 included studies. (Expanded during revision to list every included study, with a dedicated PFA platform column and an explicit synthesis-role indication).

Study	Design	Population	PFA platform	Strategy	Follow-up	Endpoint handling and role in synthesis
Verma (34)(PULSED AF)	Prospective pivotal single-arm trial	Persistent AF (*n* = 150)	Pentaspline (FARAPULSE/FARAWAVE)	PVI-only	Approx. 12 months	Composite effectiveness; descriptive only
Schiavone (31)(ATHENA)	Prospective 9-center registry	PerAF, 21.7% LSPAF (*n* = 249)	Pentaspline (FARAPULSE)	PVI-only/PVI + LAPWI/redo	Approx. 9–12 months	Study-level recurrence endpoint; descriptive only
Turagam (43)(MANIFEST-PF substudy)	Registry substudy	Persistent AF (*n* = 547)	Pentaspline (FARAPULSE)	PVI-only vs. PVI + LAPWI	12 months	KM arrhythmia-free endpoint; robustness displays + descriptive
Bianchini (17)	Comparative cohort	LSPAF (*n* = 93)	Pentaspline (FARAPULSE)	Hybrid-convergent RF vs. PFA (PVI + LAPWI)	12 months	Raw recurrence counts used for direct comparison; PFA arm in exact-count pooling
Della Rocca (32)	Single-arm cohort	PerAF >6 months/LSPAF (*n* = 72)	Pentaspline (FARAPULSE)	Staged extensive substrate PFA	Approx. 12–15 months	Single-procedure success; narrative only
Hirokami (24) (EU-PORIA)	Registry	Persistent AF (*n* = 448)	Pentaspline (FARAPULSE)	PVI-only vs. PVI plus additional substrate	12 months	KM endpoint; robustness displays + descriptive
Reddy (37) (ADVANTAGE AF Phase 1)	Pivotal single-arm IDE study	Persistent AF (*n* = 339)	Pentaspline (FARAWAVE)	PVI + PWA	12 months	Composite KM effectiveness; descriptive only (rationale in Results)
Reddy (42) (ADVANTAGE AF Phase 2) (42)	Prospective multicenter single-arm with continuous ICM	Persistent AF (*n* = 255; CTI subcohort *n* = 141)	Pentaspline (FARAWAVE) + focal-linear (FARAPOINT; CTI)	PVI + PWA ± CTI	12 months (continuous ICM)	KM/ICM-derived endpoints; descriptive only (incorporated at revision)
Chorin (19)	Single-center cohort	First-ablation persistent AF (*n* = 109)	Pentaspline (FARAPULSE)	PVI + PWI	Approx. 12–16 months	Exact 12-month event-free counts used
Pannone (14)	Head-to-head comparative study	Index persistent AF (*n* = 160)	Pentaspline (FARAPULSE)	PVI + LAPWI with PFA vs. cryoballoon	12 months	Raw recurrence counts used; safety double-zero study; exact-count pooling
Isenegger (15)	Comparative cohort	Persistent AF (*n* = 220)	Pentaspline (FARAPULSE)	PVI-only with PFA vs. cryoballoon	Approx. 12 months	Raw recurrence counts used
Ahmed (16)	Comparative cohort	Persistent AF (*n* = 200)	Pentaspline-based workflow; platform not explicitly specified	PVI + PWI with PFA vs. RFA	12 months	Percentage-derived recurrence counts (from Figure 1); direct comparison + PFA-arm pooling
Li (18)	Prospective comparative cohort	Persistent/LSPAF (*n* = 170)	Pentaspline (FARAWAVE, CARTO-guided)	EGM-guided PFA vs. anatomical PVI + PWI	12 months	Single-study direct comparison; EGM arm in exact-count pooling
Zaher (22)	Single-center cohort	Persistent AF (*n* = 93)	Multielectrode pentaspline	Beyond-PV/extensive PFA	12 months	Exact event-free counts used
Kordic (20)	Single-center cohort	Persistent/LSPAF (*n* = 94)	Pentaspline (FARAWAVE)	PVI + PWA	Approx. 12–13 months	Exact event-free counts used
Chen (21)	Prospective single-center cohort	Persistent AF (*n* = 32)	Not explicitly specified	PVI + LAPWI + CTI + mitral isthmus	12 months	Exact event-free counts used (added to Table 1 at revision)
Reddy (33) (PersAFOne)	Early prospective platform study	Persistent AF	Pentaspline (FARAPULSE)	PVI + posterior wall	12 months	Feasibility and 1-year outcomes; descriptive only
Turagam (35) (PULSE-EU)	First-in-human single-arm	Mixed AF; persistent completer subgroup (*n* = 20)	Spherical array (Globe PFA, Kardium)	PVI ± posterior wall	12 months	Persistent-subgroup KM outcome; descriptive only
Chaumont (36) (initial French experience)	Prospective multicenter	Mixed AF (*n* = 311; persistent 69.7%)	Pentaspline (FARAPULSE)	PVI ± LAPWI	12 months	Descriptive only
Lemoine (38)	Multicenter real-world cohort	Mixed AF (*n* = 138; persistent subgroup)	Pentaspline (FARAPULSE)	PVI-only	Short-term	Acute safety/short-term outcomes; descriptive only
Gunawardene (25)	Single-center cohort	Persistent AF (index PVI + LAPWI)	Pentaspline (FARAPULSE)	PVI + LAPWI vs. PVI-only	12 months	Robustness display + descriptive
Davong (30)	Prospective single-center	Persistent AF (*n* = 45)	Pentaspline (FARAPULSE)	PVI + posterior wall + mitral isthmus	Short-term (approx. 3–4 months)	Acute feasibility/safety; descriptive only

AF, atrial fibrillation; AAD, antiarrhythmic drug; CTI, cavotricuspid isthmus; EGM, electrogram; ICM, insertable cardiac monitor; IDE, investigational device exemption; KM, Kaplan–Meier; LAPWI, left atrial posterior wall isolation; LSPAF, long-standing persistent atrial fibrillation; PFA, pulsed-field ablation; PerAF, persistent atrial fibrillation; PVI, pulmonary vein isolation; PWA, posterior wall ablation; PWI, posterior wall isolation; redo, repeat ablation. Follow-up values originally reported in days were standardized as approximate months for display only.

### Statistical analysis

Formal pairwise meta-analysis was performed only when at least two clinically homogeneous head-to-head studies addressed the same comparator and outcome; single-study comparisons are reported narratively without pooled estimates. Comparative efficacy outcomes were analyzed using RevMan 5.4 (Cochrane Collaboration) to calculate risk ratios (RR) with 95% confidence intervals (CI). For rare safety events such as major complications, risk difference (RD) with 95% CI was used for pooling to preserve the contribution of double-zero event studies to the overall null evidence; Pannone 2025 (0/80 vs. 0/80) was included in the RD model.

Exploratory single-arm pooling was conducted using R (version 4.5.0) and the metaprop function from the meta package, applying logit transformation, inverse-variance method, DerSimonian–Laird random-effects model, and 0.5 continuity correction. Heterogeneity was quantified using *I*^2^ statistics, with *I*^2^ > 50% considered substantial heterogeneity. Because of the small number of studies in each comparison, formal assessment of publication bias (e.g., funnel plots or Egger's test) was not performed. Single-arm strategy estimates were not statistically compared with one another; they are non-comparative descriptive summaries and any numerical difference between strategy tiers is hypothesis-generating only.

For the comparison of PFA vs. other non-PFA strategies, we did not pool across fundamentally different comparators (RFA vs. hybrid-convergent) into a single effect estimate, as these address distinct clinical questions. Instead, we reported subgroup-specific effects separately.

## Results

### Literature search and study selection

The PRISMA flow diagram is presented in Figure 2 (updated during revision). Our search strategy identified 4,426 records across all databases. After removing duplicates, 2,418 records underwent title and abstract screening. Of these, 31 full-text articles were assessed for eligibility, and 21 studies were initially included in the systematic review. During revision, an updated verification search (August 17, 2026) formally re-evaluated two additional reports. ADVANTAGE AF Phase 2 (42), previously excluded as a companion report of the pivotal ADVANTAGE AF study, was re-assessed and formally incorporated into descriptive synthesis, yielding 22 included studies. The MANIFEST-US safety survey (43) was identified during screening but remained excluded at full-text review because PerAF/LSPAF-specific outcomes were not separately extractable (mixed paroxysmal/persistent population); given its scale, it is nonetheless discussed as contextual real-world safety evidence. The number of studies contributing to each quantitative synthesis varied by specific outcome.

**Figure 2:**
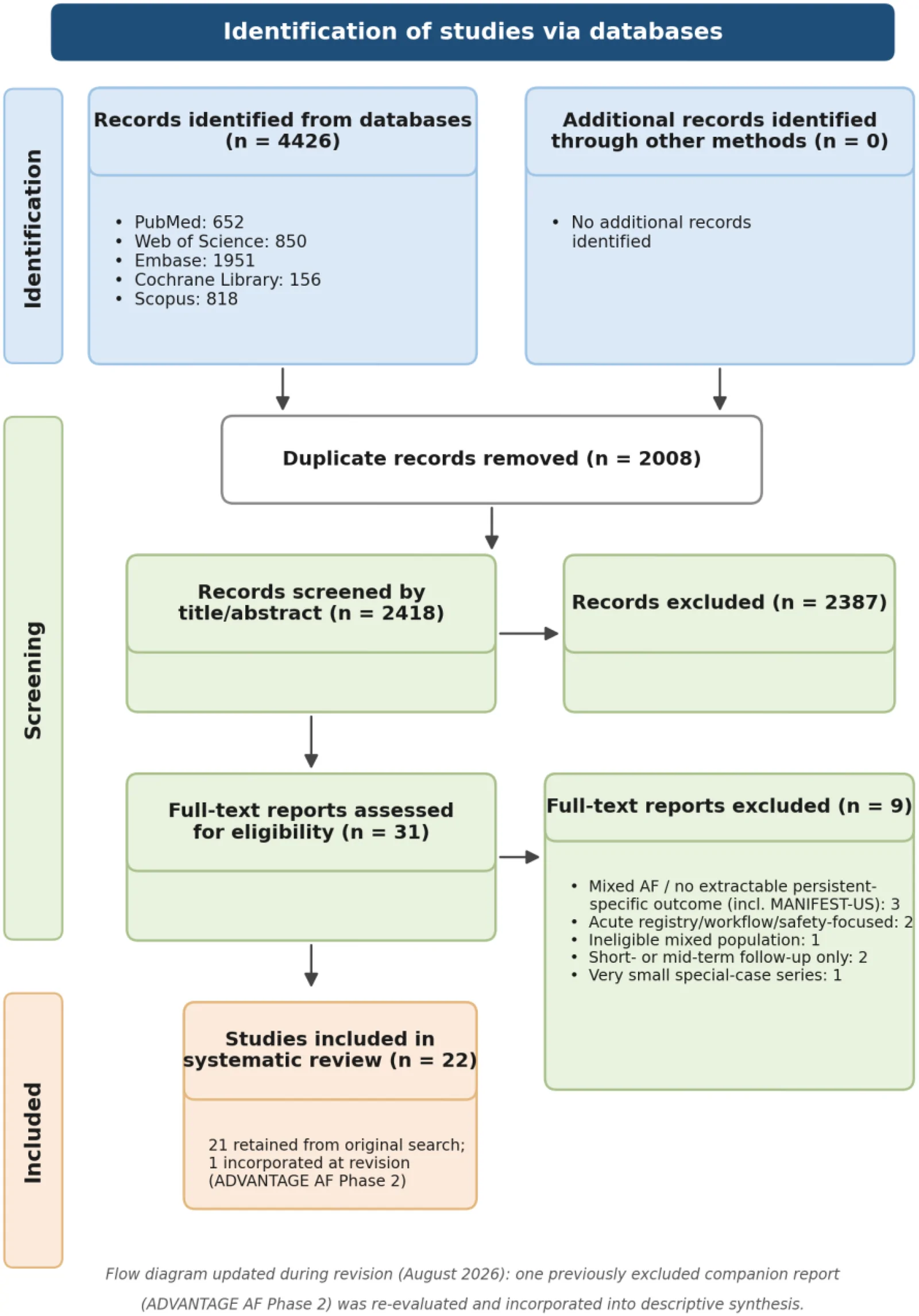
22 studies entered the systematic review (21 from the original search plus ADVANTAGE AF phase 2 incorporated during revision), and studies contributing to quantitative synthesis varied by outcome. The flow diagram was updated during revision.

The 22 included studies comprised prospective single-arm trials (*n* = 5), prospective comparative cohorts (*n* = 5), retrospective comparative studies (*n* = 3), registry-based analyses (*n* = 6), and single-center cohort studies (*n* = 3). Twelve studies contributed to quantitative syntheses (direct comparisons, exact-count single-arm pooling, or exploratory robustness displays), and ten contributed to descriptive synthesis only. In response to peer review, Table 1 has been expanded to list all 22 included studies, with the role of each study in the synthesis explicitly indicated.

### Study characteristics

Baseline characteristics of the included studies are summarized in Table 1. The total number of patients across included studies was approximately 3,100. Mean or median age ranged from 58 to 68 years, and the proportion of male participants ranged from 55% to 78%. Left atrial diameter ranged from 40 to 48 mm and left ventricular ejection fraction ranged from 52% to 62% across studies.

PFA strategies varied substantially: PVI-only, PVI plus posterior wall isolation/ablation (PWI/LAPWI/PWA), and beyond-PV or extensive substrate ablation. Follow-up duration was standardized to approximately 12 months for display purposes, though original reporting ranged from 9 to 16 months. The standard 90-day blanking period was used in most studies, though some employed 60- or 120-day blanking periods. Regarding energy platforms, twenty of the 22 studies used pentaspline PFA systems (FARAPULSE/FARAWAVE, Boston Scientific); PULSE-EU used a spherical-array catheter (Globe PFA, Kardium); and the cavotricuspid isthmus subcohort of ADVANTAGE AF Phase 2 additionally used a focal-linear PFA catheter (FARAPOINT, Boston Scientific). The platform was not explicitly specified in the published reports of Ahmed 2026 and Chen 2025. A structured qualitative comparison of platforms is provided below and in Supplementary Table S4.

### Efficacy outcomes: direct comparisons

PFA vs. cryoballoon ablation. Two studies [Pannone (14), Isenegger (15)] provided head-to-head data on 12-month atrial arrhythmia recurrence (14, 15). The exploratory pooled analysis suggested a lower risk of recurrence with PFA compared with cryoballoon ablation (RR 0.71, 95% CI 0.51–0.98; *I*^2^ = 0%; Figure 3; Table 2). However, this estimate rests on only two studies (380 patients) with markedly imbalanced statistical weights and different lesion sets [PVI-only in Isenegger (15) vs. PVI plus posterior wall isolation in Pannone (14)], so the pooled result is dominated by the more heavily weighted study. Notably, in Isenegger (15)'s own time-to-event analysis, the difference did not reach statistical significance (recurrence-free survival 72% vs. 60%; log-rank *P* = 0.079). We therefore present this pooled estimate as a preliminary, hypothesis-generating comparative signal (GRADE certainty: very low) rather than definitive evidence of superiority.

**Figure 3:**
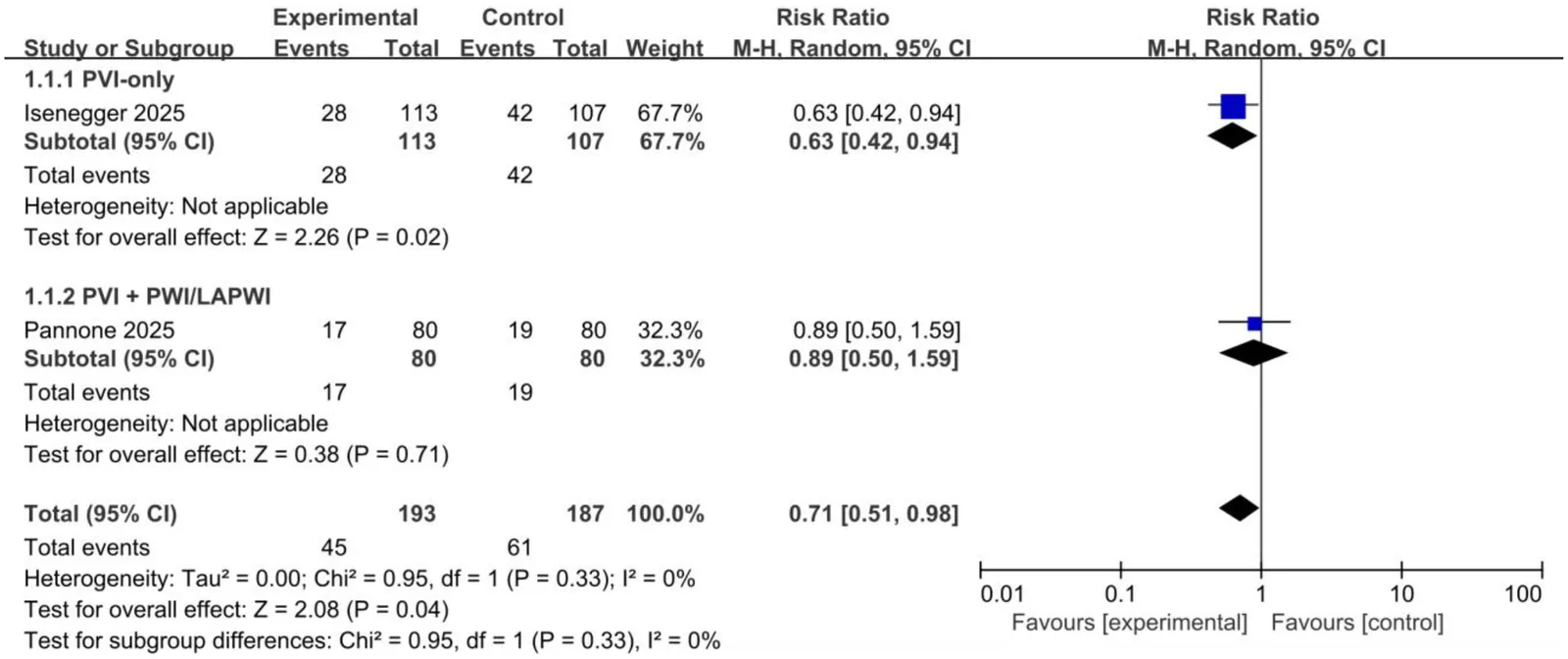
Forest plot of 12-month atrial arrhythmia recurrence: PFA versus cryoballoon ablation. This is the only multi-study direct comparative forest plot retained in the main manuscript. it is based on two studies with imbalanced weights and different lesion sets and is interpreted as a preliminary signal (GRADE: very low certainty).

**Table 2:** Direct comparative results presented in the main text.

Comparison	Presentation	Estimate (95% CI)	Interpretation
PFA vs. cryoballoon recurrence	Exploratory pooled RR (2 studies)	0.71 (0.51–0.98)	Lower recurrence with PFA; preliminary signal from two studies with imbalanced weights and different lesion sets; GRADE very low certainty; Figure 3
PFA vs. cryoballoon major complications	Pooled RD	0.00 (−0.02 to 0.02)	No statistically detectable absolute risk difference (not proof of equivalence; only 5 events); double-zero Pannone (14) retained under RD; Supplementary Figure S2
PFA vs. RFA [Ahmed (16)] recurrence	Narrative single-study comparison	RR 0.65 (0.45–0.94)	Favored PFA in this comparator setting; recurrence 30/100 vs. 46/100 derived by complementing Figure 1 12-month freedom-from-AT values (70/100 vs. 54/100)
PFA vs. hybrid-convergent [Bianchini (17)] recurrence	Narrative single-study comparison	RR 1.11 (0.67–1.86)	No clear benefit of PFA in this comparator setting
PFA vs. hybrid-convergent major complications	Narrative single-study comparison	RR 0.09 (0.00–1.47)	Major complications numerically lower with PFA; very imprecise
Within-PFA recurrence [Li (18)]	Narrative single-study comparison	RR 0.52 (0.30–0.89)	Lower recurrence with EGM-guided PFA; hypothesis-generating
Within-PFA major complications [Li (18)]	Narrative single-study comparison	RR 3.00 (0.12–72.62)	No stable safety difference due to very rare events

RR, risk ratio; RD, risk difference; EGM, electrogram-guided; PFA, pulsed-field ablation; RFA, radiofrequency ablation. For non-PFA comparator studies, subgroup-specific effects are shown without an across-subgroup pooled estimate.

a Red text marks wording revised during review.

**Table 3:** Strategy-based single-arm synthesis.

Strategy tier	Included cohorts	Pooled estimate	Heterogeneity	Interpretation
PVI-only	Not pooled	Not pooled	Not applicable	Endpoint definitions and AAD handling insufficiently harmonized; narrative only
PVI + PWI/LAPWI/PWA efficacy	Chorin (19); Pannone (14) PFA arm; Bianchini (17) PFA arm; Ahmed (16) PFA arm (percentage-derived); Kordic (20); Chen (21)	0.6600 (0.5550–0.7522)	I² = 79.0%	Exploratory pooled 12-month event-free proportion; Figure 4A
PVI + PWI/LAPWI/PWA safety	Chorin (19); Pannone (14) PFA arm; Bianchini (17) PFA arm; Kordic (20); Chen (21)	0.0133 (0.0050–0.0348)	I² = 0%	Exploratory pooled major-complication proportion; Figure 4B
Beyond-PV/extensive efficacy	Li (18) EGM arm; Zaher (22)	0.8260 (0.7630–0.8748)	I² = 0%	Exploratory pooled 12-month event-free proportion; Figure 4C
Beyond-PV/extensive safety	Li (18) EGM arm; Zaher (22)	0.0090 (0.0018–0.0435)	I² = 0%	Exploratory pooled major-complication proportion; Figure 4D

AAD, antiarrhythmic drug; LAPWI, left atrial posterior wall isolation; PVI, pulmonary vein isolation; PWA, posterior wall ablation; PWI, posterior wall isolation. Only cohorts with directly extractable exact event counts or explicitly percentage-derived counts with clear denominators were entered into the pooled single-arm analyses. Ahmed (16) was retained as a percentage-derived exact-count dataset (recurrence obtained by complementing Figure 1 freedom-from-AT values, 70/100 vs. 54/100).

a Pooled single-arm estimates are non-comparative and were not statistically compared across strategy tiers (added at revision).

PFA vs. other non-PFA strategies. These single-study comparisons are presented narratively without pooled estimates. Ahmed 2026 compared PFA with radiofrequency ablation (RFA) in 200 patients with persistent AF, both incorporating posterior wall isolation (16); PFA was associated with lower recurrence risk (RR 0.65, 95% CI 0.45–0.94), noting that the recurrence counts were derived by complementing the 12-month freedom-from-AT percentages reported in the study's Figure 1 (70/100 vs. 54/100) and are therefore percentage-derived rather than directly tabulated raw counts. In contrast, Bianchini (17) compared PFA with hybrid-convergent ablation in 93 patients with LSPAF (17) and found no clear advantage of PFA (RR 1.11, 95% CI 0.67–1.86). Because these two single studies used fundamentally different comparators (endocardial RFA vs. hybrid thoracoscopic/epicardial convergent procedure), we did not pool them. The forest plot in Supplementary Figure S1 displays these subgroup-specific effects separately without cross-subgroup pooling.

Within-PFA strategy comparison. Li (18) provided single-study evidence comparing electrogram-guided PFA with anatomical PVI + PWI in 170 patients with persistent or LSPAF (18). Electrogram-guided PFA was associated with lower recurrence risk (RR 0.52, 95% CI 0.30–0.89). This single-study finding is hypothesis-generating and requires confirmation in larger, multi-center studies.

### Safety outcomes: direct comparisons

PFA vs. cryoballoon ablation. For major complications, we re-pooled data using risk difference (RD) to retain the double-zero event study (Pannone: 0/80 vs. 0/80) (14). No statistically detectable difference in major complication rates was observed between PFA and cryoballoon (RD 0.00, 95% CI −0.02 to 0.02; Supplementary Figure S2). Because only five events occurred in total (3/193 vs. 2/187), this finding indicates an absence of a demonstrable difference; it does not establish equivalence between the two strategies.

PFA vs. hybrid-convergent ablation. Bianchini (17) reported numerically lower major complications with PFA vs. hybrid-convergent ablation, but the event rate was very low and the estimate imprecise (RR 0.09, 95% CI 0.00–1.47; Supplementary Figure S4) (17). This single-study result is descriptive and does not support a formal safety conclusion.

Within-PFA safety comparison. Li (18) reported no stable safety difference between electrogram-guided and anatomical PFA strategies (RR 3.00, 95% CI 0.12–72.62) due to very rare events (Supplementary Figure S5) (18).

### Studies identified or re-evaluated during revision

#### ADVANTAGE AF phase 2 (incorporated into descriptive synthesis)

ADVANTAGE AF Phase 2 (42) is a prospective multicenter study of 255 patients with PerAF undergoing PVI plus posterior wall ablation with the pentaspline PFA catheter, with a subcohort (*n* = 141) additionally receiving cavotricuspid isthmus ablation using a focal-linear PFA catheter under nitroglycerin prophylaxis, and with continuous insertable cardiac monitor (ICM) follow-up. Freedom from atrial arrhythmia at 12 months was 73.4% when the ICM data were used to emulate traditional intermittent monitoring, and 52.0% for freedom from any atrial arrhythmia ≥30 s under continuous monitoring; the adverse event rate was 2.4%, with no pulmonary vein stenosis, esophageal fistula, or phrenic nerve palsy. Because its endpoints are Kaplan–Meier-based and ICM-derived and cannot be converted to exact 12-month event counts, the study contributes to descriptive synthesis only; its results are directionally consistent with the present pooled estimates.

#### MANIFEST-US (identified; excluded from formal synthesis; contextual safety evidence)

MANIFEST-US (43) is a retrospective safety survey of 41,968 patients (36.8% persistent AF) treated with the pentaspline PFA catheter across 102 US centers. Major adverse events occurred in 0.63% (cardiac tamponade 0.16%, vascular injury requiring intervention 0.18%, stroke 0.10%, coronary artery spasm 0.10%, acute renal failure requiring dialysis 0.02%), with no esophageal fistula, persistent phrenic nerve paralysis, or pulmonary vein stenosis; a rare signal of unexplained early sudden death/cardiac arrest (0.019%) was noted. Because PerAF/LSPAF-specific 12-month rhythm and safety outcomes were not separately extractable, the study met the pre-specified exclusion criterion for formal synthesis; it is discussed as contextual real-world safety evidence in the Discussion.

#### Why ADVANTAGE AF (phase 1) was not included in the pooled PVI + PWI single-arm analysis

ADVANTAGE AF (37) is one of the largest prospective studies of PFA in persistent AF (*n* = 339, PVI plus posterior wall ablation), and we agree it is central to this literature. It was not entered into the exact-count PVI + PWI single-arm pooling because its primary effectiveness endpoint is composite (atrial arrhythmia recurrence, redo ablation, cardioversion, or AAD escalation) and Kaplan–Meier-based, and raw 12-month event counts corresponding to our harmonized rhythm endpoint were not extractable; under our pre-specified endpoint rules, composite IDE endpoints were retained for descriptive synthesis only. Its reported freedom from documented atrial arrhythmia recurrence (65.8% for any atrial arrhythmia; 71.2% for AF) is nevertheless directionally consistent with the pooled PVI + PWI estimate (66.0%). The same rule applies to ADVANTAGE AF Phase 2.

### Single-arm strategy synthesis

PVI-only studies were not subjected to overall single-arm pooling because endpoint definitions, AAD management approaches, and availability of precise counts were insufficiently harmonized to support credible uniform synthesis. These studies were integrated narratively (Table 3).

PVI plus posterior wall isolation (PWI/LAPWI/PWA). Six cohorts with precisely extractable raw 12-month counts were included (14, 16, 17, 19–21). Exploratory single-proportion meta-analysis showed a pooled freedom from atrial arrhythmia of 66.0% (95% CI 55.5%–75.2%; I²=79.0%; Figure 4A) and a pooled major complication rate of 1.33% (95% CI 0.50%–3.48%; *I*^2^ = 0%; Figure 4B). The substantial heterogeneity in efficacy likely reflects varying patient substrates, AAD management, and monitoring intensity across cohorts. The Ahmed 2026 PFA-arm data were percentage-derived from the study's Figure 1, as noted above.

**Figure 4:**
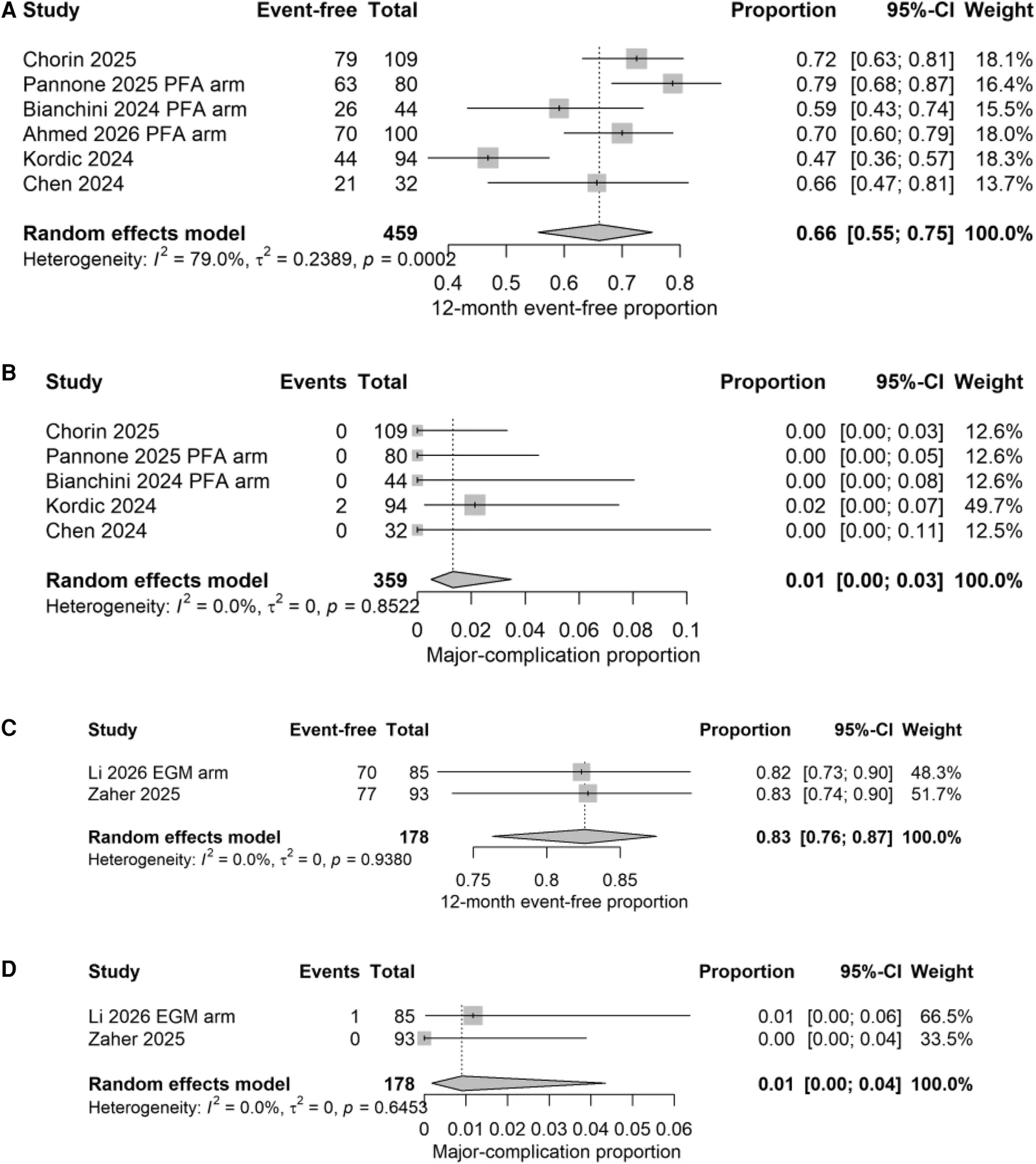
**(A)** Exploratory pooled 12-month event-free proportion for PVI + PWI/LAPWI/PWA cohorts. (Non-comparative, hypothesis-generating estimate). **(B)** Exploratory pooled major-complication proportion for PVI + PWI/LAPWI/PWA cohorts. **(C)** Exploratory pooled 12-month event-free proportion for beyond-PV/extensive cohorts. (Two cohorts only; non-comparative, hypothesis-generating estimate). **(D)** Exploratory pooled major-complication proportion for beyond-PV/extensive cohorts.

Beyond-PV/extended substrate ablation. Two cohorts with exact counts were available [Zaher (22), Li (18) EGM arm]. The pooled freedom from atrial arrhythmia was 82.6% (95% CI 76.3%–87.5%; *I*^2^ = 0%; Figure 4C) and the pooled major complication rate was 0.90% (95% CI 0.18%–4.35%; *I*^2^ = 0%; Figure 4D). These two single-arm estimates (82.6% vs. 66.0%) were not statistically compared and derive from different, non-randomized cohorts; they are strictly non-comparative and hypothesis-generating. The absence of heterogeneity in the beyond-PV tier should be interpreted cautiously given the small number of cohorts.

### PFA platform distribution and qualitative comparison

Nearly all PerAF/LSPAF evidence included in this review derives from pentaspline PFA systems. Pentaspline catheters (FARAPULSE/FARAWAVE) deliver biphasic, bipolar pulses through a configurable flower/basket multielectrode array and have validated posterior wall workflows; the spherical-array catheter (Globe PFA, Kardium) integrates mapping and ablation in a single-shot 122-electrode array, but its persistent-AF evidence remains limited to the small PULSE-EU subgroup; the focal-linear catheter (FARAPOINT) has so far been used mainly as an adjunct for cavotricuspid isthmus ablation within ADVANTAGE AF Phase 2. Waveform characteristics, lesion depth, catheter-tissue contact requirements, posterior wall workflows, and collateral-tissue effects differ across these platforms, so they cannot necessarily be treated as one interchangeable intervention. A structured qualitative comparison is provided in Supplementary Table S4. Because quantitative subgroup analysis by platform was not feasible (too few non-pentaspline studies), our pooled results should be read as pertaining predominantly to pentaspline PFA; generalizability to other platforms requires dedicated study.

### Quality assessment and certainty of evidence

NOS scores for comparative cohorts in the primary analysis ranged from 6/9 to 7/9. Modified 7-item tool scores for single-arm and registry studies ranged from 5/7 to 6/7. The most common limitations were non-random treatment allocation, residual confounding despite comparable baseline characteristics, and composite efficacy endpoints rather than raw recurrence counts. The quality assessment plot is shown in Figure 1, with study-level scoring detailed in Table 4. GRADE certainty ratings were low to very low for all key outcomes (Supplementary Table S3), driven by the non-randomized designs, the small number of studies per comparison, indirectness from differing lesion sets, and imprecision from few events.

**Table 4:** Quality scores for the main-study set.

Study	Tool	Score	Main limitation
Isenegger (15)	NOS	7/9	Non-randomized treatment allocation
Pannone (14)	NOS	7/9	Retrospective comparator selection
Ahmed (16)	NOS	7/9	Residual confounding despite comparable baseline characteristics
Bianchini (17)	NOS	7/9	Comparator differed fundamentally from standard endocardial thermal ablation
Li (18)	NOS	6/9	Single-center non-randomized strategy allocation
Verma (34)	Modified 7-point	6/7	Composite effectiveness rather than raw recurrence counts
Schiavone (31)	Modified 7-point	6/7	Mixed strategy registry with limited exact-count harmonization
Turagam 2024	Modified 7-point	6/7	Registry design and comparator heterogeneity
Della Rocca (32)	Modified 7-point	5/7	Selected extensive-ablation cohort and no exact 12-month raw count
Hirokami (24)	Modified 7-point	6/7	Registry design with non-randomized extra-PV allocation
Reddy 2025	Modified 7-point	6/7	Composite effectiveness endpoint
Chorin (19)	Modified 7-point	6/7	Single-center protocolized cohort
Zaher (22)	Modified 7-point	5/7	Single-center extensive strategy cohort
Kordic (20)	Modified 7-point	5/7	Redo-heavy real-world cohort

NOS, newcastle-ottawa scale. The modified 7-point tool is detailed in Supplementary Table S1. Scores were used to contextualize risk of bias and interpretability rather than to weight pooled effects directly.

### Exploratory robustness displays

In the previous version these analyses were labeled “sensitivity analyses”; we have relabeled them exploratory robustness displays and describe their composition explicitly, because they pool registry-based within-PFA strategy comparisons rather than test the robustness of the main comparative meta-analysis. Supplementary Figure S6 pools two registry-based safety comparisons of additional posterior wall/substrate ablation vs. PVI-only (Hirokami 2025: 1/101 vs. 7/347; Turagam 2024: 3/131 vs. 6/416; overall RR 1.11, 95% CI 0.35–3.50; *I*^2^ = 0%) (23, 24). Supplementary Figure S7 adds Gunawardene (25) to the within-PFA recurrence comparison (index PVI + LAPWI vs. PVI-only) (25); the direction of effect was unchanged. These displays are presented for completeness only, and no firm conclusions are drawn from them.

## Discussion

### Principal findings

This systematic review and meta-analysis of PFA in PerAF and LSPAF yields three principal findings, all supported by low to very low certainty evidence. First, in an exploratory two-study pooled analysis, PFA was associated with lower 12-month atrial arrhythmia recurrence than cryoballoon ablation (RR 0.71, 95% CI 0.51–0.98); given that this estimate derives from only two studies with imbalanced weights and different lesion sets, it is best regarded as the strongest available—but still preliminary and very-low-certainty—comparative signal, requiring confirmation in adequately powered randomized trials. Second, no statistically detectable difference in major complications was observed between PFA and cryoballoon, with risk difference pooling preserving the contribution of the double-zero study; this reflects absence of a demonstrable difference, not proven equivalence. Third, exploratory, non-comparative single-arm analyses yielded a numerically higher pooled event-free proportion for beyond-PV/extended substrate strategies than for PVI plus posterior wall strategies (82.6% vs. 66.0%); because these estimates come from different, non-randomized cohorts and were never compared head-to-head, they are strictly hypothesis-generating.

Our hybrid analytical framework—combining direct comparative meta-analysis, where clinically homogeneous study pairs exist, with narrative synthesis of single-study comparisons and exploratory single-arm strategy synthesis—was designed to make the inherent heterogeneity of the PerAF/LSPAF PFA literature transparent rather than to eliminate it. We deliberately did not pool across incomparable comparators or heterogeneous endpoint definitions; nevertheless, we acknowledge that even within strata, the number of contributing studies is small, and the certainty of every pooled estimate remains low to very low.

The trial landscape has evolved rapidly. The BEAT PERS-AF trial, presented as Late-Breaking Science at EHRA 2026, is among the first randomized comparisons of PFA vs. radiofrequency ablation specifically in persistent AF, employing uniform PVI plus posterior line ablation (44). The PIFPAF-PFA trial is the first randomized superiority trial to test whether adding PFA-based posterior wall isolation to PVI reduces atrial tachyarrhythmia recurrence in persistent AF, using continuous implantable cardiac monitor follow-up (45). In addition, the PFA-SHAM trial provides the first placebo-controlled evidence for PFA efficacy and quality-of-life benefit (46), while the larger FACIL-AF randomized study supports PFA's favorable efficacy and safety profile relative to cryoballoon (47). The PVI-SHAM-AF trial, presented at ESC Congress 2026, provides important sham-controlled methodological context for catheter ablation overall, though it is not PFA-specific (48). Because these trials were published or presented after our primary search cutoff (April 18, 2026) or employed endpoints not harmonizable with our pre-specified 12-month exact-count framework, they are presented here as contextual evidence rather than formally incorporated into the 22-study quantitative synthesis.

The incorporation of these recently published or presented randomized trials reinforces the principal findings while adding important nuance. PIFPAF-PFA indicates that adjunctive PWI should not be assumed to provide a large recurrence benefit, but also leaves open a smaller effect that the trial was not powered to detect. BEAT PERS-AF provides randomized evidence that PFA achieves at least comparable 12-month effectiveness to RF ablation under uniform lesion-set conditions, with a marked safety advantage. PFA-SHAM provides placebo-controlled evidence that PFA's efficacy is not attributable to placebo effects. FACIL-AF supports the broader PFA-vs.-cryoballoon signal with randomized data from a larger cohort, albeit in a less selected AF population.

### Comparison with previous evidence

Our finding that PFA is associated with lower recurrence than cryoballoon in PerAF/LSPAF is consistent with the broader PFA literature, though previous mixed-AF population reviews reported less stable recurrence advantages (1–4). We believe this apparent discrepancy reflects our more focused research question and stratified analytical approach. Early meta-analyses by Aldaas et al. emphasized shorter procedure times without significant recurrence differences (1), while subsequent reviews by Amin et al. and de Campos et al. began observing potential PFA advantages in AF recurrence, alongside reduced esophageal injury but rising cardiac tamponade signals (2, 3). The review by Iqbal et al. supported the overall assessment of “faster procedure, no significant difference in overall complications, unstable recurrence advantage” (4). The newly incorporated ADVANTAGE AF Phase 2 results (42) are directionally consistent with our pooled estimates and uniquely demonstrate, through continuous ICM monitoring, how strongly monitoring intensity affects the measured recurrence rate (73.4% freedom when emulating intermittent monitoring vs. 52.0% under continuous monitoring), reinforcing the endpoint- and monitoring-related caveats that run through our analysis.

The BEAT PERS-AF trial (44) adds a critical randomized data point to the PFA-vs.-RFA comparison in persistent AF. Unlike Ahmed 2026 (16), which was a single-center comparative cohort, BEAT PERS-AF employed a multicenter randomized design with uniform PVI plus posterior line ablation in both arms. Its finding of 65% effectiveness for PFA under an A'Hern performance-goal design, alongside the marked safety disparity (no serious PFA complications vs. 12% PV stenosis, 3% esophageal ulcer, and 6% vascular complications with RF), provides the most rigorous comparative safety evidence yet for PFA in persistent AF. This safety advantage is mechanistically consistent with PFA's myocardial selectivity and aligns with the absence of esophageal injury and pulmonary vein stenosis in our pooled safety analysis and in the large MANIFEST-US registry (43).

The PIFPAF-PFA trial (45) therefore requires a cautious, non-binary interpretation. For the primary endpoint of first atrial tachyarrhythmia recurrence during days 91–365, recurrence occurred in 51/102 patients (50.6%) with PVI + PWI vs. 63/104 (60.6%) with PVI alone (RR 0.75, 95% CI 0.51–1.09; *P* = .13). Thus, statistical superiority was not demonstrated. However, early separation of the Kaplan–Meier curves and this approximately 10-percentage-point absolute numerical difference, corresponding to an approximately 25% relative risk reduction, raise the possibility of a smaller treatment effect than anticipated. The trial was powered to detect a 33.3% relative (20% absolute) risk reduction and was therefore not designed to establish or exclude a smaller benefit. In addition, mean total arrhythmia burden was lower with PWI (6.9% vs. 11.0%; between-group difference −4.1 percentage points, 95% CI −8.0 to −0.2; *P* = .04), and recurrences with longer minimum episode durations were generally less frequent. Because the secondary endpoints were not adjusted for multiplicity, these findings are hypothesis-generating. Thus, PIFPAF-PFA argues against assuming a large universal benefit from routine PFA-based PWI, but it does not exclude a modest benefit in selected patients. This interpretation is consistent with CAPLA (10) and randomized-trial-focused meta-analyses (7, 8), and it reinforces that the exploratory 66.0% vs. 82.6% single-arm comparison cannot establish superiority of broader ablation targets.

The PFA-SHAM trial (46) provides the first placebo-controlled evidence specifically for PFA. Its dramatic efficacy signal (6.7% vs. 83.3% recurrence) and quality-of-life improvement (AFEQT +43.9 vs. +11.3 points) confirm that the recurrence reductions observed in open-label PFA studies are not attributable to placebo effects. However, its 6-month endpoint, small sample size (*n* = 60), and mixed paroxysmal/persistent population limit direct extrapolation to our 12-month PerAF-specific syntheses. The larger PVI-SHAM-AF trial (48), presented at ESC Congress 2026, provides important methodological context: in that double-blind, sham-controlled study of 262 patients using various energy sources, catheter ablation reduced AF recurrence by 20% absolute vs. sham but did not significantly improve the primary quality-of-life endpoint at 6 months. We emphasize that PVI-SHAM-AF is not PFA-specific and should not be conflated with PFA-SHAM; rather, both trials underscore that sham-controlled designs are essential for delineating true treatment effects from placebo effects in AF ablation, and that PFA-SHAM's positive findings specifically validate PFA efficacy.

FACIL-AF (47) supports the PFA-vs.-cryoballoon efficacy signal with the largest randomized dataset to date (*n* = 269), reporting 86.6% vs. 77.8% 12-month freedom from recurrence (*p* < 0.001) and significantly fewer periprocedural complications with PFA (0.7% vs. 8.1%, *p* = 0.0053). The lower pulmonary vein reconnection rate with PFA (22.2% vs. 46.0%) suggests more durable acute isolation, which may contribute to the observed recurrence advantage. Because FACIL-AF's success rates are consistent with a predominantly paroxysmal AF population and PerAF-specific outcomes were not separately reported, it is not incorporated into our PerAF-specific cryoballoon comparison; nevertheless, it strengthens the overall PFA efficacy and safety narrative.

By narrowing the analysis to PerAF/LSPAF and separating comparisons by comparator type (cryoballoon, RFA, hybrid-convergent), our study provides a more clinically relevant evidence summary. The observation that PFA outperformed cryoballoon but showed no clear advantage over hybrid-convergent ablation is not contradictory—it reflects that these comparators address fundamentally different clinical scenarios (endocardial energy modality comparison vs. hybrid surgical/epicardial approach), and both observations rest on very few studies.

Regarding posterior wall strategies, our findings align with the nuanced picture emerging from the thermal ablation literature. Shrestha et al. supported PVI + LAPWI for improving rhythm outcomes (6), whereas Ibrahim et al. and Li et al. found no consistent benefit and potential harm (increased AT/AFL) (7, 8). The CAPLA trial's negative results for routine PWI in first-ablation persistent AF (10) further support that posterior wall strategy effects depend on patient selection, energy modality, and implementation quality rather than being universally beneficial or ineffective.

### Interpretation of extended substrate results

The numerically higher exploratory event-free proportion observed for beyond-PV/extended substrate strategies (82.6% vs. 66.0% for PVI + PWI) must not be interpreted as evidence that broader ablation targets improve outcomes, and we have removed or qualified any language implying “higher success” throughout the manuscript. Several critical caveats apply. First, this signal derives from only two precise-count cohorts, both from highly experienced, specialized centers with likely stringent patient selection, whereas the PVI + PWI estimate derives from six heterogeneous cohorts (*I*^2^ = 79%). Second, the two strategy tiers were never compared head-to-head, and no between-tier statistical comparison was performed or should be inferred; differences in AF duration, left atrial substrate, previous ablation, AAD management, monitoring intensity, operator expertise, and patient selection could entirely explain the numerical difference (confounding by indication).

Pranata et al.'s focused meta-analysis on PFA-LAPWI demonstrated extremely high technical success rates but no significant recurrence reduction compared with PVI-only (12). Their meta-regression further suggested that left atrial diameter (a marker of atrial myopathy burden) was more predictive of recurrence than lesion set extent—each 1 mm increase associated with approximately 6% higher ATa recurrence probability (12). This supports our interpretation that patient substrate burden may be more determinative of long-term outcomes than simply “drawing additional circles.” The PIFPAF-PFA trial (45) provides the most direct randomized evidence on this question to date. By randomizing persistent AF patients to PFA-based PVI with vs. without posterior wall isolation, and by employing continuous ICM monitoring in all patients, PIFPAF-PFA minimized both confounding by indication and ascertainment bias. Its prespecified primary endpoint was not met, but the trial was powered for a larger effect than may plausibly exist and showed numerical and secondary-outcome signals favoring PWI, including lower total arrhythmia burden and fewer longer sustained episodes. Accordingly, the trial argues against routine, unselected PWI based on an assumed large benefit, while leaving open the possibility of a smaller benefit in appropriately selected patients. This supports our interpretation that patient substrate burden—reflected by left atrial diameter and atrial myopathy (12)—may be more determinative of long-term outcomes than simply adding ablation targets, and that any benefit from extended substrate modification likely requires rigorous patient selection and assurance of lesion completeness rather than universal application.

Furthermore, Banai et al. suggested that PFA posterior wall isolation is less likely to cause restrictive left atrial physiology than thermal posterior wall isolation (26), but incomplete or marginal extensive ablation may itself create macro-reentrant substrate (8). Thus, extended substrate PFA should be understood as a “strategy requiring rigorous patient selection and ensuring lesion quality,” not a universally reproducible fixed protocol.

### Safety considerations

Our safety analysis yielded three important insights. First, for rare events like major complications, risk difference (RD) pooling is preferable to risk ratio (RR) pooling when double-zero event studies exist, as RD preserves the “no difference” information carried by large sample sizes. Our RD analysis showed no significant difference between PFA and cryoballoon (RD 0.00, 95% CI −0.02 to 0.02), consistent with the absolute event pattern (3/193 vs. 2/187). However, with only five events in total, this result indicates an absence of a statistically detectable difference and must not be interpreted as evidence of equivalence; safety conclusions should remain cautious.

Second, PFA's safety profile is better understood as an altered complication spectrum rather than risk elimination, and important PFA-specific complications deserve separate summary. Multiple reviews consistently indicate lower esophageal and phrenic nerve injury risk with PFA (1–4), consistent with its myocardial selectivity, and no esophageal fistula, persistent phrenic paralysis, or pulmonary vein stenosis was reported in the 41,968-patient MANIFEST-US survey (43). However, cardiac tamponade signals have not disappeared (0.16% in MANIFEST-US; also noted in prior reviews 2–4, 43), and vascular complications requiring intervention (0.18%) and stroke (0.10%) remain the most frequent major events in real-world use (43). Emerging PFA-specific concerns include intraoperative hemolysis related to lesion number and catheter-tissue contact (27), hemoglobinuria-induced acute kidney injury (28) (dialysis-requiring renal failure 0.02% in MANIFEST-US 43), and coronary artery spasm—reported as reversible events with mitral isthmus applications [Davong (30)], in 0.10% of MANIFEST-US procedures, and addressed with nitroglycerin prophylaxis in ADVANTAGE AF Phase 2 (42, 43). A rare signal of unexplained early sudden death or cardiac arrest (0.019%) in MANIFEST-US warrants continued post-market surveillance (43). The BEAT PERS-AF trial (44) provides randomized comparative safety data for PFA vs. RF in persistent AF. Under uniform PVI plus posterior line ablation conditions, PFA was associated with no serious complications, whereas RF was associated with pulmonary vein stenosis >50% in 12%, esophageal ulcer in 3%, and vascular access complications in 6%. Together with the absence of esophageal fistula, persistent phrenic paralysis, and pulmonary vein stenosis in MANIFEST-US (43), these findings suggest a low incidence of these collateral-tissue complications with pentaspline PFA; they do not establish complete elimination of these risks or safety equivalence across PFA platforms and operator experience.

Third, cerebrovascular safety requires particular caution. In a prospective registry of 4,221 consecutive AF ablation procedures at a high-volume US center, Ferro et al. (49) found higher 30-day stroke/TIA with PFA than with RFA [10/2,144 [0.47%] vs. 2/2,077 [0.10%]; weighted risk difference 0.36%, 95% CI 0.03%–0.70%; *P* = 0.03], while other procedural complications were low and similar between groups. The absolute event rate was low, and exploratory mediation analyses did not identify more extensive ablation as an independent explanation. Nevertheless, this propensity-weighted single-center signal—distributed across operators and the adoption period—underscores that myocardial selectivity does not eliminate periprocedural embolic risk and warrants dedicated post-market surveillance (49).

This finding is not necessarily inconsistent with recent randomized-evidence syntheses suggesting that AF ablation reduces stroke over longer follow-up. In an 18-RCT meta-analysis of 5,877 patients, Providencia et al. (50) reported lower stroke risk with catheter ablation than medical therapy (RR 0.63, 95% CI 0.45–0.87). Similarly, Montané et al. (51) found that catheter ablation reduced ischemic stroke occurring more than 30 days after treatment (RR 0.63, 95% CI 0.43–0.92), while noting a marked early periprocedural increase in ischemic stroke at or before 30 days (RR 6.81, 95% CI 1.56–29.8); consequently, the overall any-ischemic-stroke estimate was not significantly reduced (RR 0.77, 95% CI 0.55–1.09). These reviews mainly evaluated conventional thermal ablation, particularly RFA and cryoballoon ablation, against medical therapy and therefore capture a potential longer-term rhythm-control benefit. In contrast, the Ferro registry directly compares early procedural safety between PFA and RFA. The most coherent interpretation is that effective AF ablation may reduce later stroke risk while PFA—like other left atrial ablation modalities—retains a small but clinically important early embolic risk requiring standardized neurologic surveillance in future studies.

Our analysis, focused on major complications with limited direct-comparison data, cannot quantitatively assess these emerging events, but acknowledging them is essential to avoid characterizing PFA as “absolutely safer”.

### Endpoint heterogeneity and its implications

We regard endpoint heterogeneity as the single most important threat to comparability in this literature, and we emphasize it prominently because it conditions every quantitative result in this review. Rhythm endpoints differ in off-AAD/on-AAD handling, blanking period duration, and whether composite efficacy definitions (incorporating AAD escalation, cardioversion, or repeat ablation) are used. Our approach prioritized off-AAD outcomes, labeled AAD-unseparated endpoints when necessary, and excluded composite IDE endpoints from precise-count pooling. This improved single-arm interpretability but meant that some PVI-only evidence could only be integrated narratively.

Monitoring intensity itself significantly affects recurrence estimates (29), as illustrated most directly by ADVANTAGE AF Phase 2, where continuous ICM monitoring nearly doubled the detected arrhythmia burden relative to emulated intermittent monitoring (42). Generalized PFA reviews often mixed “atrial arrhythmia recurrence,” “AF recurrence,” and “composite treatment failure” (1–4), while PerAF/LSPAF studies more frequently incorporated posterior wall, mitral isthmus, or extensive substrate strategies (12, 23, 30–32). By insisting on blanking-period-adjusted, approximately 12-month rhythm recurrence endpoints and distinguishing off-AAD from AAD-unseparated status, we aimed to maximize comparability while acknowledging residual heterogeneity.

### PFA platform considerations

Pentaspline, spherical-array, focal-linear, and lattice-tip PFA technologies differ in waveform characteristics, lesion depth, contact requirements, posterior wall workflows, and collateral-tissue effects, and cannot necessarily be treated as one intervention. Because the PerAF/LSPAF evidence base is overwhelmingly pentaspline-based (20 of 22 studies), our findings—including the comparative signal against cryoballoon and the single-arm strategy estimates—should be read primarily as pentaspline-PFA evidence. Outcomes with spherical-array, lattice-tip, and focal-linear platforms in PerAF/LSPAF require dedicated prospective reporting, and future studies should consistently identify the platform, waveform, and lesion workflow used (Supplementary Table S4).

### Limitations

This study has several limitations. First, most included studies were non-randomized, introducing potential selection bias and confounding. Second, the number of studies in each direct comparison was small (2–3 studies), limiting statistical power and precluding formal publication bias assessment, specifically, the PFA-vs.-cryoballoon pooled estimate rests on two studies with imbalanced weights and different lesion sets (PVI-only vs. PVI plus posterior wall isolation), is dominated by the more heavily weighted study, and is rated very-low certainty by GRADE. Third, PFA technology is rapidly evolving, with multiple catheter platforms (pentaspline, spherical array, lattice) that may have different safety and efficacy profiles; our analysis did not stratify by catheter type due to insufficient data. Fourth, single-arm strategy synthesis is inherently susceptible to confounding by indication, and our beyond-PV results are based on only two cohorts from experienced centers. Fifth, long-term outcomes beyond 12 months were sparsely reported, limiting assessment of durability. Sixth, the review protocol was registered retrospectively (INPLASY202640082) after the search had been completed, which does not provide the safeguards of prospective registration. Seventh, for one study [Ahmed (16)], recurrence counts were percentage-derived from a published figure rather than directly tabulated. Eighth, the rapidly evolving PFA trial landscape means that additional sham-controlled and randomized comparative data continue to emerge. The trials discussed as contextual evidence in this Discussion—BEAT PERS-AF (44), PIFPAF-PFA (45), PFA-SHAM (46), FACIL-AF (47), and PVI-SHAM-AF (48)—were published or presented after our primary search cutoff or employed endpoints not harmonizable with our pre-specified framework, precluding formal inclusion in the 22-study quantitative synthesis. Future updates of this review may need to incorporate additional trials as they are published. Finally, our primary search cutoff (April 18, 2026) may miss very recent studies, although the updated verification search performed during revision (August 17, 2026) identified no further studies that would alter the conclusions. Ninth, the post-search prospective registry by Ferro et al. (49) signals the need for continued cerebrovascular surveillance and was incorporated narratively rather than into the prespecified quantitative synthesis.

### Future directions

The existing literature does not support reducing PFA research in PerAF/LSPAF to simple questions of “energy substitution” or “whether to add posterior wall isolation.” A more productive framework recognizes that PFA may have altered safety boundaries and lesion achievement modalities, making certain strategies previously limited by esophageal risk more implementable (6–8, 12), but that long-term efficacy still depends on patient substrate, lesion sets, lesion completeness, and endpoint consistency (29). It should also be noted that the early RF randomized study by Kim suggested that adjunctive posterior wall isolation might improve outcomes (39), while subsequent cryoballoon-related prospective studies by Ahn (2022) and Aryana (2021) also provided relatively positive signals in persistent atrial fibrillation (40, 41).

Based on this framework, future research should: (1) conduct adequately powered RCTs comparing PFA with cryoballoon and RFA in PerAF/LSPAF with uniform lesion sets, standardized off-AAD endpoints, and prespecified monitoring protocols; (2) prospectively validate the incremental value of PVI-only, posterior wall strategies, and extended substrate approaches across different atrial myopathy phenotypes; (3) report platform- and waveform-specific outcomes, given the evolving PFA technology landscape and the current pentaspline dominance of the evidence base; (4) incorporate continuous rhythm monitoring (as piloted in ADVANTAGE AF Phase 2) to make recurrence ascertainment comparable across trials; and (5) establish longer-term follow-up data to evaluate durability beyond 12 months. (6) Conduct sham-controlled trials specifically of PFA in PerAF/LSPAF with 12-month endpoints to confirm whether the dramatic 6-month efficacy and quality-of-life signals observed in PFA-SHAM (46) are sustained in this more challenging population.

## Conclusions

In patients with persistent and long-standing persistent atrial fibrillation, current evidence suggests efficacy and an overall favorable but still evolving safety profile for PFA, with low to very low certainty for key outcomes. The randomized PIFPAF-PFA trial did not demonstrate statistically significant superiority of posterior wall isolation add-on therapy, while leaving open a smaller-than-anticipated benefit that the trial was not powered to detect; together with BEAT PERS-AF's randomized safety comparison favoring PFA over radiofrequency ablation under uniform lesion-set conditions, these data underscore the need for larger, adequately powered randomized trials. An exploratory two-study pooled analysis suggests lower 12-month atrial arrhythmia recurrence with PFA vs. cryoballoon ablation—an encouraging but preliminary comparative signal requiring confirmation with uniform lesion sets. No statistically detectable difference in major complications was found, which should be interpreted as absence of demonstrable difference rather than equivalence. Numerically higher event-free proportions with extended substrate strategies are non-comparative and hypothesis-generating only and cannot establish that broader ablation targets improve outcomes. Future research should prioritize prospective, randomized comparisons with standardized off-AAD endpoints, continuous rhythm monitoring, standardized neurologic surveillance, stratification by atrial myopathy phenotype, and platform-specific reporting to clarify the optimal lesion set for individual patients.

## Data Availability

The original contributions presented in the study are included in the article/Supplementary Material, further inquiries can be directed to the corresponding author.
