# Supplementary material for: Efficacy, safety, and strategy-modifying factors of pulsed field ablation for persistent and long-standing persistent atrial fibrillation: a systematic review and meta-analysis
Source: Front Cardiovasc Med. 2026 Oct 5;13:1877204. doi: 10.3389/fcvm.2026.1877204 (PMC13677292; doi:10.3389/fcvm.2026.1877204)
Supplement: Supplementary file 1 [file Datasheet1.docx]

**Supplementary Appendix**

**PRISMA 2020 Checklist and Full Search Strategies**

**Review title:** Efficacy, Safety, and Strategy-Modifying Factors of Pulsed Field Ablation for Persistent and Long-Standing Persistent Atrial Fibrillation: A Systematic Review and Meta-analysis

Searches were run from database inception to 18 April 2026. The strategies below were harmonized into submission-ready syntax consistent with the review scope (PFA in persistent/long-standing persistent AF).

**Supplementary Appendix 1. PRISMA 2020 Checklist**

| **Section/Topic** | **Item** | **Checklist item** | **Location reported** |
| --- | --- | --- | --- |
| TITLE | 1 | Identify the report as a systematic review and meta-analysis. | Title |
| ABSTRACT | 2 | Provide a structured summary of background, methods, results, and conclusions. | Abstract |
| INTRODUCTION | 3 | Describe the rationale for the review in the context of existing knowledge. | Introduction, paragraphs 1–5 |
| INTRODUCTION | 4 | State the objectives or questions addressed by the review. | Introduction, final paragraph |
| METHODS | 5 | Specify inclusion and exclusion criteria for studies and reports. | Methods, Eligibility and endpoint harmonization rules |
| METHODS | 6 | Specify all information sources and the date each source was last searched. | Methods, Search strategy; Supplementary Appendix 2 |
| METHODS | 7 | Present the full search strategies for all databases and sources. | Supplementary Appendix 2 |
| METHODS | 8 | Describe methods used to decide study eligibility, including reviewer workflow. | Methods, Search strategy |
| METHODS | 9 | Describe methods used to collect data from reports, including reviewer workflow. | Methods, Search strategy |
| METHODS | 10a | List and define all outcomes sought. | Methods, Eligibility and endpoint harmonization rules; Major complications definition |
| METHODS | 10b | List and define other variables sought. | Methods, Search strategy |
| METHODS | 11 | Specify the methods used to assess risk of bias in included studies. | Methods, Quality assessment |
| METHODS | 12 | Specify the effect measures used for each outcome. | Methods, Statistical analysis |
| METHODS | 13a | Describe the process used to decide which studies were eligible for each synthesis. | Methods, Eligibility and endpoint harmonization rules |
| METHODS | 13b | Describe any methods required to prepare data for presentation or synthesis. | Methods, Eligibility and endpoint harmonization rules; Statistical analysis |
| METHODS | 13c | Describe methods used to tabulate or visually display results. | Results; Tables 1–4; Figures 1–4 |
| METHODS | 13d | Describe synthesis methods and statistical models. | Methods, Statistical analysis |
| METHODS | 13e | Describe methods used to explore heterogeneity. | Methods, Statistical analysis; Results; Supplementary Figures S6–S7 |
| METHODS | 13f | Describe any sensitivity analyses. | Results, sensitivity displays in Supplementary Figures S6–S7 |
| METHODS | 14 | Describe methods used to assess risk of bias due to missing results (reporting bias). | Methods, Statistical analysis (not formally assessed because of few studies) |
| METHODS | 15 | Describe any methods used to assess certainty or confidence in the body of evidence. | Not performed |
| RESULTS | 16a | Describe the results of the search and selection process. | Results, Figure 1 |
| RESULTS | 16b | Cite studies that might appear eligible but were excluded, and explain why. | Figure 1 |
| RESULTS | 16c | Cite each included study and present its characteristics. | Results; Table 1 |
| RESULTS | 17 | Present risk-of-bias assessments for included studies. | Results; Table 4; Figure 4; Supplementary Table S1 |
| RESULTS | 18 | Present results of all individual studies for all outcomes. | Results; Table 2; Supplementary Figures S3–S5 |
| RESULTS | 19 | Present results of all syntheses undertaken. | Results; Tables 2–3; Figures 2–3; Supplementary Figures S1–S2, S6–S7 |
| RESULTS | 20 | Present assessments of risk of bias due to missing results. | Not formally assessed because of few studies |
| RESULTS | 21 | Present assessments of certainty/confidence in the body of evidence. | Not assessed |
| DISCUSSION | 22 | Provide an overall interpretation of the results. | Discussion |
| DISCUSSION | 23a | Discuss limitations of the evidence included in the review. | Discussion, sections 2–10 |
| DISCUSSION | 23b | Discuss limitations of the review processes used. | Discussion, sections 2–5 and 10 |
| DISCUSSION | 23c | Discuss implications of the results for practice, policy, and future research. | Discussion, sections 11–13; Conclusion |
| OTHER INFORMATION | 24a | Provide registration information for the review. | Methods, Search strategy (not registered) |
| OTHER INFORMATION | 24b | Indicate where the review protocol can be accessed, or state that none was prepared. | No public protocol |
| OTHER INFORMATION | 24c | Describe and explain amendments to information provided at registration or in the protocol. | Not applicable |
| OTHER INFORMATION | 25 | Describe sources of financial or non-financial support. | To be completed per target journal requirements |
| OTHER INFORMATION | 26 | Declare competing interests. | To be completed per target journal requirements |
| OTHER INFORMATION | 27 | Report availability of data, code, and other materials. | To be completed per target journal requirements |

**Supplementary Appendix 2. Full Database Search Strategies**

• Concept blocks were combined using database-specific syntax. Search fields were limited to title/abstract/keywords where appropriate. No outcome filter was applied.

• Core concept A: pulsed field ablation / electroporation terms.

• Core concept B: atrial fibrillation terms.

• Core concept C: persistent, long-standing persistent, or non-paroxysmal disease terms.

**PubMed (searched through 18 April 2026)**

(("Pulsed Field Ablation"[tiab] OR "pulse field ablation"[tiab] OR "pulsed-field ablation"[tiab] OR "irreversible electroporation"[tiab] OR electroporation[tiab] OR Farapulse[tiab] OR FARAWAVE[tiab] OR pentaspline[tiab]) AND ("Atrial Fibrillation"[Mesh] OR "atrial fibrillation"[tiab]) AND (persistent[tiab] OR "long-standing persistent"[tiab] OR "long standing persistent"[tiab] OR non-paroxysmal[tiab] OR nonparoxysmal[tiab] OR PerAF[tiab] OR LSPAF[tiab]))

**Web of Science Core Collection**

TS=(("pulsed field ablation" OR "pulse field ablation" OR "pulsed-field ablation" OR "irreversible electroporation" OR electroporation OR Farapulse OR FARAWAVE OR pentaspline) AND ("atrial fibrillation") AND (persistent OR "long-standing persistent" OR "long standing persistent" OR non-paroxysmal OR nonparoxysmal OR PerAF OR LSPAF))

**Embase (Elsevier)**

('pulsed field ablation':ti,ab,kw OR 'pulse field ablation':ti,ab,kw OR 'pulsed-field ablation':ti,ab,kw OR 'irreversible electroporation':ti,ab,kw OR electroporation:ti,ab,kw OR farapulse:ti,ab,kw OR farawave:ti,ab,kw OR pentaspline:ti,ab,kw) AND ('atrial fibrillation'/exp OR 'atrial fibrillation':ti,ab,kw) AND (persistent:ti,ab,kw OR 'long-standing persistent':ti,ab,kw OR 'long standing persistent':ti,ab,kw OR non-paroxysmal:ti,ab,kw OR nonparoxysmal:ti,ab,kw OR peraf:ti,ab,kw OR lspaf:ti,ab,kw)

**Scopus**

TITLE-ABS-KEY(("pulsed field ablation" OR "pulse field ablation" OR "pulsed-field ablation" OR "irreversible electroporation" OR electroporation OR Farapulse OR FARAWAVE OR pentaspline) AND ("atrial fibrillation") AND (persistent OR "long-standing persistent" OR "long standing persistent" OR non-paroxysmal OR nonparoxysmal OR PerAF OR LSPAF))

**Cochrane Library (CENTRAL)**

(("pulsed field ablation" OR "pulse field ablation" OR "pulsed-field ablation" OR "irreversible electroporation" OR electroporation OR Farapulse OR FARAWAVE OR pentaspline):ti,ab,kw) AND (("atrial fibrillation"):ti,ab,kw) AND ((persistent OR "long-standing persistent" OR "long standing persistent" OR non-paroxysmal OR nonparoxysmal OR PerAF OR LSPAF):ti,ab,kw)

**Manual search**

Reference lists of included studies and relevant review articles were screened manually to identify additional eligible reports.
